# High-Quality Draft Genome Sequence of *Bifidobacterium longum* E18, Isolated from a Healthy Adult

**DOI:** 10.1128/genomeA.01084-13

**Published:** 2013-12-19

**Authors:** Daria Zhurina, Alexey Dudnik, Mark S. Waidmann, Verena Grimm, Christina Westermann, Katrin J. Breitinger, Jing Yuan, Douwe van Sinderen, Christian U. Riedel

**Affiliations:** Institute of Microbiology and Biotechnology, University of Ulm, Ulm, Germanya; Institute of Plant Biology, University of Zurich, Zurich, Switzerlandb; Institute of Disease Control and Prevention, Academy of Military Medical Sciences, Beijing, Chinac; Alimentary Pharmabiotic Centre and Department of Microbiology, Biosciences Institute, University College Cork, Cork, Irelandd

## Abstract

Bifidobacteria are important gastrointestinal commensals of a number of animals, including humans, and various beneficial effects on host health have been attributed to them. Here, we announce the noncontiguous finished genome sequence of *Bifidobacterium longum* E18, isolated from a healthy adult, which reveals traits involved in its interaction with the host.

## GENOME ANNOUNCEMENT

Bifidobacteria are considered to be GRAS (generally recognized as safe) microorganisms that are widely used as probiotics. Probiotics are defined as live microbial food supplements that have a beneficial effect on host health when administered in adequate amounts (1). The beneficial effects of bifidobacteria have been studied mainly in animal models and include immune modulation, pathogen inhibition, and the alleviation of intestinal inflammation (2–7). Genome sequencing and analysis of members of the genus *Bifidobacterium* are powerful approaches to identify the genetic determinants involved in the interaction with the host. To extend knowledge of this subject, we have sequenced the genome of the commensal *B. longum* E18 strain, which was isolated from the feces of a healthy adult.

A 7.9-kb paired-end library was constructed and sequenced using a Roche Genome Sequencer FLX Titanium platform by a commercial sequencing service provider, Eurofins MWG Operon (Ebersberg, Germany). A total of 246,462 individual quality-filtered reads comprising 53,491,261 bp were obtained, resulting in a 26.7-fold average coverage. The read sequences were assembled using Newbler 2.3 and Staden package 2.0.0b9 into six contigs put together into one megascaffold. Initial tRNA, rRNA, and open reading frame (ORF) prediction and functional annotation were done using the RAST server (8). The translational start sites of automatically annotated ORFs were manually corrected using Artemis software (9) based on the positions of potential ribosomal binding sites, the G+C profile, and alignments with homologous ORFs from other organisms. Manual corrections of automatically assigned functions were verified on an individual gene-by-gene basis using BLASTp (10) searches against the nonredundant protein sequence database.

The estimated genome size of strain E18 is 2,372,966 bp, with an average G+C content of 59.96%. The genome contains 1,862 protein-encoding genes, 4 rRNA operons, and an unusually high number of tRNA genes (62 in total). Putative functions were assigned to 1,299 genes (67%). The amino acid sequences encoded by 99 genes contain putative signal peptide sequences, and thus, these proteins might be secreted via the classical *sec* pathway. *B. longum* E18 harbors a complete *tatABC* secretion system (BLONG_0088-90) and at least 4 of the secreted proteins (BLONG_0091, BLONG_0223, BLONG_0425, and BLONG_1620) are predicted to be exported via this pathway. Approximately half of all secreted proteins are predicted hydrolases, substrate binding proteins, and components of transport systems with a putative role in the acquisition of nutrients, which represents an adaptation of bifidobacteria to the gastrointestinal habitat (11–13). Further genes with a potential role in interaction with the host carried in the *B. longum* E18 genome include two putative exopolysaccharide (EPS) biosynthesis gene clusters (BLONG_0398-0417 and BLONG_2008-2021) and a gene cluster encoding Tad pili (BLONG_0140-0145). Both traits were previously shown to be important for *in vivo* colonization and modulation of the immune system by bifidobacteria (2, 14).

### Nucleotide sequence accession numbers.

The genome sequence of *B. longum* E18 has been deposited at DDBJ/EMBL/GenBank under the accession no. AUYD00000000. The version described in this paper is version AUYD01000000. The assigned NCBI taxonomy identification number is 1322347.
